# Environmental Literacy: Knowledge for a Healthier Public

**DOI:** 10.1289/ehp.115-a494

**Published:** 2007-10

**Authors:** Ron Chepesiuk

In 1988, New York City’s West Harlem community had a problem. The recently opened North River Sewage Treatment Plant, which stretches eight blocks along the Hudson River, was doing a poor job of processing about 170 million gallons of raw sewage daily. Residents were concerned about the foul smells coming from the plant, and parents complained that their children were suffering from respiratory problems. The community knew it needed help, but it also needed something else: information on the exposures it was facing, on the health effects of those exposures, and on the courses of action open to the people. When the community mobilized months later to form West Harlem Environmental Action Inc. (WE ACT), it had taken the first step toward cultivating just that sort of environmental literacy.

Within six years, WE ACT had reached a settlement with the treatment plant. But the learning is still going on. In 2000 WE ACT began partnering with the NIEHS-funded Columbia Center for Children's Environmental Health to promote environmental education and literacy among the 600,000 adults and children in Northern Manhattan. Among other efforts, WE ACT and the NIEHS center have partnered with the Lang Youth Medical Project, a six-year science education and mentoring program for middle school students that takes advantage of the educational resources of the Columbia University Medical Center. The project’s mission is to inspire, motivate, and support young people in the Washington Heights area of New York City so they can realize their college and career aspirations in the health sciences. Nada Hamade, a research associate at the NIEHS center, estimates that since partnering, WE ACT and the center have educated at least 60,000 people from the Northern Manhattan area about environmental health issues through a variety of means—youth meetings, conferences, forums, leadership training, and outreach campaigns.

The WE ACT–NIEHS partnership is a prime example of the environmental literacy movement at work in the United States. The movement involves a wide variety of federal, state, and private groups nationwide that have mobilized and developed dynamic programs to educate people about the environment and its relevance to human health. Its ultimate goal: to help people develop skills they can use to make responsible and wise decisions about the environment and environmental health.

Each day people make decisions that affect the environment, whether they are getting ready to go to work, preparing dinner, or buying products for the house or garden. It’s imperative, then, that the public learn and understand how their actions and lifestyle intersect with the environment.

Environmental educators believe that the earlier children begin to learn about sound stewardship principles, the better it is for them, their families, and society. “Today’s children will one day be responsible for making decisions that will shape the future health of the environment,” wrote Deborah Mitchell, senior editor for *Environmental Protection* magazine, in “Promote Environmental Education for Children,” an article appearing on http://www.charityguide.org/. “To prepare them for such responsibilities, they need a sound environmental education as a foundation upon which to make those decisions.” Most important, says Mitchell, environmental literacy helps develop and expand children’s critical thinking skills, prepares them for citizenship, nurtures their appreciation of the natural world, and enhances their physical well-being.

“Environmental literacy seeks to change human behavior so that humanity can create a sustainable and environmentally friendly quality of life,” explains Christina Zarcadoolas, an associate clinical professor in the Department of Community and Preventive Medicine at the Mount Sinai School of Medicine. “To do that, people need a wide range of skills that can help them understand, assess, and use environmental health information.”

## More Than Science

“Studying the environment is not all about science,” explains M. Jane Teta, spokesperson for the Environmental Literacy Council and principal health scientist for the New York City–based Exponent Inc., a consultancy of engineers and health scientists. “We think it’s important that learners understand the environment from all perspectives and that they do it critically before they develop a position. [The Environmental Literacy Council’s] approach is to present an environmental issue and then show that it has different stakeholders. We encourage students to role-play so they get the opportunity to see how people view things.”

The Environmental Literacy Council has partnered with the National Science Teachers Association (NSTA) to create several new professional development modules that incorporate environmental issues into the context of the “Earth, life, and the physical sciences” National Science Education Standard, part of a rubric for best teaching practices. Each module includes background information and provides recommendations for further reading, information about online training resources, and suggestions for activities that can foster and enhance classroom discussion. The modules are available on the Environmental Literacy Council website at http://www.enviroliteracy.org/.

Grassroots Environmental Education, a Port Washington, New York–based nonprofit dedicated to educating the public about the links between common environmental exposures and human health, has developed several literacy projects that are being used in the Port Washington school district. The Safe Lawn Flag Project, for instance, focuses on educating school children in grades 3 through 6 on issues relating to pesticides and the available alternatives to their use. “We try to show students that everything they do has an impact on the environment, whether they are flushing the toilet or going to the grocery store with their parent,” explains Patti Wood, executive director of Grassroots Environmental Education. “We want to make them understand that all human action has environmental consequences. Often, [these consequences] are negative, and humans must address them.”

*EHP* also offers help for teachers trying to integrate environmental health information into their classrooms. The *EHP* Science Education website at http://www.ehponline.org/science-ed-new/ provides lessons developed specifically for high school students around *EHP* news articles. The lessons demonstrate that environmental health concepts can be incorporated into a variety of subject areas, including many nonscience areas such as geography, language arts, history, government/civics, and communications.

Environmental literacy proponents don’t seek to provide any particular “right” answer in studying environmental issues. Instead, they seek to instruct learners through self-discovery and the acquisition of problem-solving skills that help them evaluate different viewpoints.

Environmental literacy initiatives that seek to educate rather than proselytize have many benefits for society as well as the individual. With the National Science Foundation’s *Science and Engineering Indicators 2006* showing that Americans get most of their health information from the media, it’s imperative that the public be better informed about the environment so they can make responsible decisions, support good public policy, and help create a sustainable natural environment.

“My research has shown that the environmental information the public gets from the media is mostly skewed in a specific way—towards the bad news,” says Seymour Garte, a professor of environmental and occupational health in the University of Pittsburgh School of Public Health and author of *Where We Stand: A Surprising Look at the Real State of the Planet*. “A lot of initiatives like the Clean Water Act and the Wildlife Protection Act have worked, but you see little evidence of that in the media,” he says. “Its slant on doom and gloom doesn’t make for a balanced view, nor does it give citizens the type of information they need to make informed decisions. That’s where the environmental literacy movement can play an important role.”

## Connecting the Dots

Environmental educators credit Rachel Carson with sparking the modern environmental movement in 1956 when she first expounded on the importance of environmental education and its characteristics at the early childhood level in her book *The Sense of Wonder*.

Carson’s pioneering work, including the 1962 publication of *Silent Spring*, led to the creation of Earth Day in 1970. Since then, millions of teachers and students have joined together each April during National Environmental Education Week, the country’s largest organized environmental event that seeks to promote Earth Day. During the 2007 National Environmental Education Week, 1,453 environmental education partners taught more than 3.5 million students about the importance of caring for the environment through a full range of activities in K–12 classrooms, zoos, nature centers, museums, and aquariums.

“National Environmental Education Week is more relevant today than it’s ever been,” says Leyla McCurdy, senior director of health and the environment at the National Environmental Education Foundation (NEEF) in Washington, DC, which the yearly event. “We still need to do a much better job showing people how they can . . . protect the environment while improving the quality of their lives.”

McCurdy believes that environmental health plays an important role in promoting the broad goals of the environmental literacy movement. “People usually relate to issues that impact on themselves and their loved ones,” she explains. “Environmental literacy, therefore, can only be truly successful when it makes the connection between health and the environment.”

Recently, NEEF launched its Children’s Environmental Health Faculty Champions Initiative to establish a network of children’s environmental health experts at medical and nursing schools throughout the country. The faculty champions are taking a leadership role in incorporating children’s environmental health into their academic institutions in a meaningful fashion, educating health professionals about health risks unique to children, teaching courses, and providing expertise and support to their surrounding communities.

McCurdy points out there are many places besides schools where environmental knowledge can be taught. The Children’s Environmental Health Faculty Champions Initiative, she says, can serve as a model for making sustainable institutional changes within a broad spectrum of professions.

Other sources believe the environmental health community must do a better job of educating people about how their personal choices in diet and consumption have not only health consequences but environmental ones well. “When I went to public school in Argentina we had a course simply called ‘hygiene,’ which taught us about the health threats to the environment,” says Mary Pearl, president of the Wildlife Trust in New York City. “In the United States, a ‘health class’ usually involves the teaching of sex education. That’s why it’s difficult for Americans to see the impact that the environment has on health.”

The Wildlife Trust is known for innovative work on protecting the intricate relationship between ecosystems and human health, but the organization also believes it’s important that its work foster environmental literacy. So it has created a children’s educational program called The Wild Ones. The program’s website displays students’ work and provides information about endangered species and the people who protect them.

“We educate children about what we call the bioscape, which is a new way of looking at the environment,” Pearl says. She explains that the bioscape is the place that communities and their residents share with other living organisms as they conduct their daily living. “The goal is to help children appreciate and understand the environmental dynamics at play in the world around them. It involves showing how wildlife and their habitat can be protected while still taking into account how humans need to use the landscape for survival.” This is increasingly important in a time when many children have less opportunity to experience the natural environment—humans spend, on average, nearly 90% of their time indoors, according to a survey in the May–June 2001 issue of the *Journal of Exposure Analysis and Environmental Epidemiology*.

## In Search of Education

Environmental health actually has an identity problem when it comes to literacy. Two literacy movements—health literacy and environmental literacy—work parallel to each other, and the twain has not necessarily met. The World Wide Web offers hundreds of health literacy websites providing valuable consumer information, but most of them do not have anything to say about how the environment impacts health. “Health literacy and environmental literacy are two different worlds,” says Devra Davis, head of the Center for Environmental Oncology at the University of Pittsburgh and author of *The Secret History of the War on Cancer*. “One of our goals is to bring the two movements together.”

Ted Schettler, executive director of the Science and Educational Health Network in Ann Arbor, Michigan, believes the health field shares some of the blame for the lack of public understanding of the health connection to the environment. “We, as health professionals, whether we are in public or clinical health, can do a better job to educate the public about the connection and the importance of environmental health on human life,” Schettler says. “We have clear data that shows people in medical school are getting poor training in environmental health literacy, and so they have a poor understanding of the effects of environmental factors on human health.”

Again, say sources, it goes back to how people are trained professionally. “Health care professionals and educators have limited understanding of the broader environment because it’s not incorporated into their education,” says Steve Heilig, director of public health and education for the San Francisco Medical Society. “The [opposite] is true, too. People educated in classic environmental programs don’t have any specialized education in health.”

The division into health literacy and environmental literacy has no doubt weakened the impact of environmental health education in the public school system, where science and health usually occupy two separate tracks. Environmental health education was first introduced in grades K through 12 in the early 1990s, and since then, many individual schools have introduced it. But it still has a far way to go to be adopted into the standard curriculum by most school districts and states [for more information, see “Setting a New Syllabus: Environmental Health Science in the Classroom,” *EHP* 112:A814–A819 (2004)].

The general field of environmental literacy has fared much better. Nearly 2.5 million K–12 teachers include some sort of environment-related science education in the classroom, according to the Environmental Literacy Council, and the majority of the students at over half of all colleges take an environment-related course. Still, these stats do not impress environmental health specialists like Heilig who see a fundamental flaw in environmental education. “The environmental education being taught in the school system from the lower and middle school grades through college is not sophisticated,” he says. “The [environmental] education, moreover, doesn’t get any better or more sophisticated as students move along through the school system.”

Augusto Medina, project manager of the Environmental Education and Training Partnership (EETAP), a teacher training program based at the University of Wisconsin–Steven’s Point, agrees with Helig. “There is no coherency or consistency in the way the environment is taught in the U.S. school system,” Medina says. “The educational system does help to make children aware of environmental issues, but it doesn’t really help them to reach the next level—that is, develop the skills they need to help them apply what they learn to the real problems in their communities. The objective of environmental education should be to develop active citizens who can deal with environmental issues.”

## National Direction

In 1990, Congress passed the National Environmental Education Act, which provides funding for teacher training, national-and local-level environmental education grants, the President’s Environmental Youth Award Program, and environmental education projects among federal agencies. The act also established the EPA Office of Environmental Education to provide national leadership in promoting environmental literacy. Ginger Potter, senior education specialist at the Office of Environmental Education, explains that the EETAP, which is funded through the EPA, is responsible for the development of standards for environmental education, including guidelines of excellence for materials, programs, and projects; learner outcomes; in-service teacher training; and nonformal education.

Each year, the EPA makes more than 200 grants totaling $2–3 million to support environmental education projects nationwide. Yet, as Potter explains, “There has been no change to the act since 1996. . . . Like all pieces of legislation, the act has to be reauthorized.”

David E. Blockstein, a senior scientist with the National Council for Science and the Environment in Washington, DC, adds, “The act badly needs updating. A lot has changed since 1990.”

The No Child Left Behind Act, another significant piece of federal legislation passed by the George W. Bush administration in 2001, has had a major impact on American education. But the impact has not necessarily been positive, according to sources who say that No Child Left Behind has marginalized environmental literacy because it focuses on math and language arts to the exclusion of other subjects, including environmental science. “No Child Left Behind has had a negative impact on science education in general,” says Stefani Hines, senior curriculum and assessment specialist and environmental health specialist with the University of New Mexico College of Pharmacy. “The attitude in the public schools has been ‘if it’s not tested, it’s not important.’”

Currently, an effort is under way in Congress to strengthen and expand environmental education in America’s classrooms. Representative John Sarbanes (D–MD) and Senator Jack Reed (D–RI) are cosponsoring the No Child Left Inside Act of 2007, which would provide federal funding to states to train teachers in environmental education, operate model environmental programs, and create environmental literacy plans.

Environmental education and respect for our natural surroundings ought to be a major focus in U.S. classes, says Sarbanes. “This legislation will make funds available to teachers and students for the establishment of innovative programs within our school curriculums,” he elaborates. “The next generation faces enormous national and international challenges. Sound environmental education will make for healthier lifestyles and provide a foundation for the next generation so they can tackle those challenges head on.”

The law stipulates that there be “cooperation” between federal agencies. However, says Potter, what that means is yet to be determined. If adopted, the legislation could conceivably abolish the EPA’s Office of Environmental Education and reestablish environmental education within the U.S. Department of Education. Yet, says Potter, “I question whether the Department of Education is the place to put environmental education. The Department of Education has never been interested in environmental education, and nobody there has any expertise in the field [of environmental science].”

However this political drama plays out, sources believe more needs to be done at the federal level to strengthen environmental education. “We need resources to create incentives at all levels,” says McCurdy. “At the moment, there is really a lack of incentive for people to get involved in environmental education. For example, in the schools, teachers need [consistent] funding and opportunity to get further education and to integrate environmental education into curricula.”

“Environmental education should be an independent part of the curriculum, not something that’s added on,” Medina says. “But that can’t be done until we have enough teachers who are trained to teach environmental education.”

Environmental educators believe that environmental literacy must be pursued through a multipronged approach that reaches out not only to teachers, parents, and students but also to politicians, community leaders, medical practitioners, and the workplace. “The level of environmental literacy in the workplace is very low,” says Angelo Garcia, an industrial hygienist with Future Environment Designs, a Syosset, New York–based company that trains companies in handling industrial waste. “We must start at the most basic level to make the workforce more environmentally literate.”

“Literacy is a never-ending process,” Hines says. “Like any form of education, environmental literacy must never stop, and it has to be promoted and encouraged at all levels and sectors. There are always new people to recruit.”

## Figures and Tables

**Figure:**
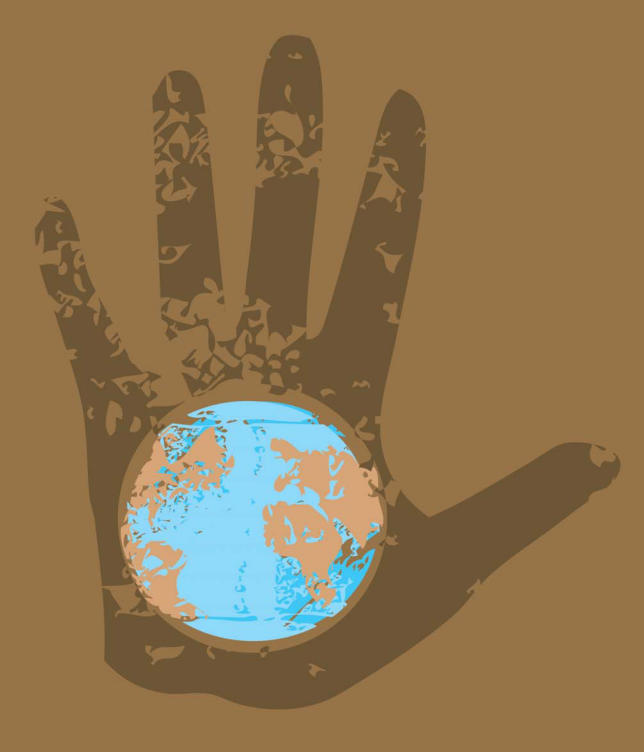


**Figure:**
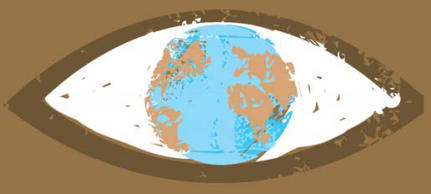
Today‘s children will one day be responsible for making decisions that will shape the future health of the environment. To prepare them for such responsibilities, they need a sound environmental education as a foundation upon which to make those decisions. –Deborah Mitchell Environmental Protection in “Promote Environmental Education for Children”

**Figure:**
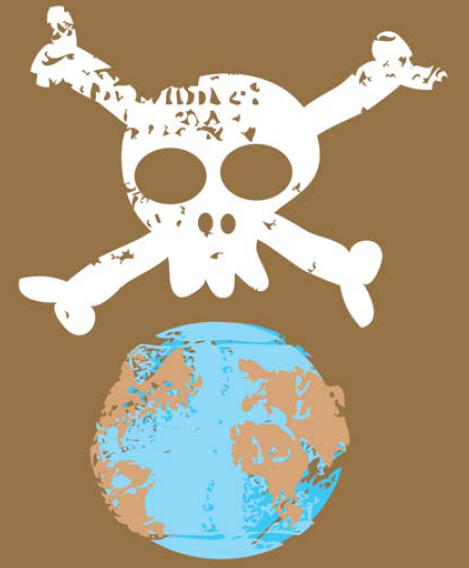
The environmental information the public gets from the media is mostly skewed in a specific way-towards the bad news. . . . Its slant on doom and gloom doesn‘t make for a balanced view, nor does it give citizens the type of information they need to make informed decisions. That‘s where the environmental literacy movement can play an important role. –Seymour Garte University of Pittsburgh School of Public Health

**Figure:**
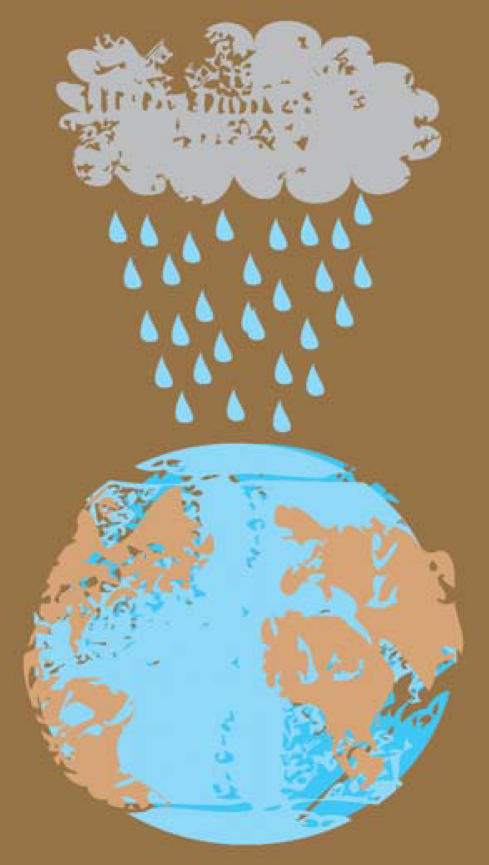
We educate children about what we call the bioscape, which is a new way of looking at the environment. The goal is to help children appreciate and understand the environmental dynamics at play in the world around them. It involves showing how wildlife and their habitat can be protected while still taking into account how humans need to use the landscape for survival. –Mary Pearl Wildlife Trust

**Figure:**
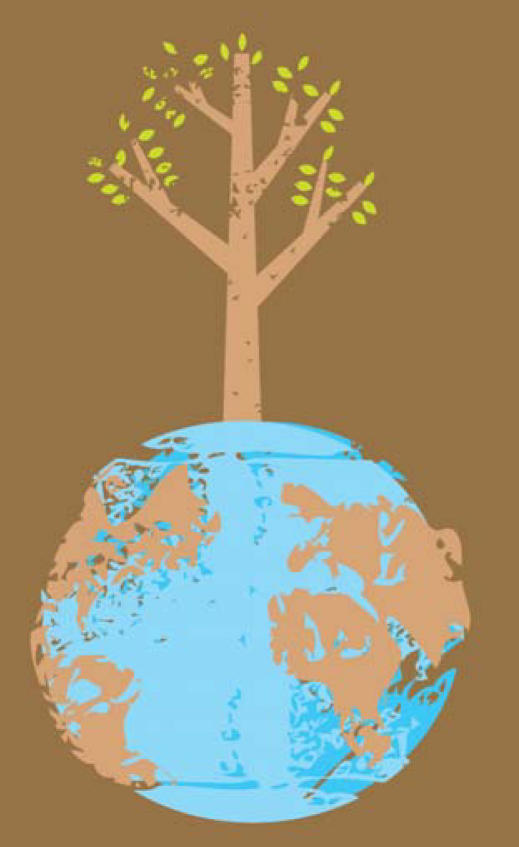
Literacy is a never-ending process. Like any form of education, environmental literacy must never stop, and it has to be promoted and encouraged at all levels and sectors. –Stefani Hines University of New Mexico College of Pharmacy

